# Characterizing Functional Connectivity Differences in Aging Adults using Machine Learning on Resting State fMRI Data

**DOI:** 10.3389/fncom.2013.00038

**Published:** 2013-04-25

**Authors:** Svyatoslav Vergun, Alok S. Deshpande, Timothy B. Meier, Jie Song, Dana L. Tudorascu, Veena A. Nair, Vikas Singh, Bharat B. Biswal, M. Elizabeth Meyerand, Rasmus M. Birn, Vivek Prabhakaran

**Affiliations:** ^1^Medical Physics, University of Wisconsin–MadisonMadison, WI, USA; ^2^Clinical Neuroengineering Training Program, University of Wisconsin–MadisonMadison, WI, USA; ^3^Radiology, University of Wisconsin–MadisonMadison, WI, USA; ^4^Electrical and Computer Engineering, University of Wisconsin–MadisonMadison WI, USA; ^5^Neuroscience Training Program, University of Wisconsin–MadisonMadison, WI, USA; ^6^Biomedical Engineering, University of Wisconsin–MadisonMadison, WI, USA; ^7^Medicine and Biostatistics, University of PittsburghPittsburgh, PA, USA; ^8^Biostatistics and Medical Informatics, University of Wisconsin–MadisonMadison, WI, USA; ^9^Radiology, New Jersey Medical SchoolNewark, NJ, USA; ^10^Waisman Center, University of Wisconsin–MadisonMadison, WI, USA; ^11^Psychiatry, University of Wisconsin–MadisonMadison, WI, USA; ^12^Psychology, University of Wisconsin–MadisonMadison, WI, USA

**Keywords:** aging, resting state fMRI, support vector machine, reorganization

## Abstract

The brain at rest consists of spatially distributed but functionally connected regions, called intrinsic connectivity networks (ICNs). Resting state functional magnetic resonance imaging (rs-fMRI) has emerged as a way to characterize brain networks without confounds associated with task fMRI such as task difficulty and performance. Here we applied a Support Vector Machine (SVM) linear classifier as well as a support vector machine regressor to rs-fMRI data in order to compare age-related differences in four of the major functional brain networks: the default, cingulo-opercular, fronto-parietal, and sensorimotor. A linear SVM classifier discriminated between young and old subjects with 84% accuracy (*p*-value < 1 × 10^−7^). A linear SVR age predictor performed reasonably well in continuous age prediction (*R*^2^ = 0.419, *p*-value < 1 × 10^−8^). These findings reveal that differences in intrinsic connectivity as measured with rs-fMRI exist between subjects, and that SVM methods are capable of detecting and utilizing these differences for classification and prediction.

## Introduction

Functional networks are defined by a temporal correlation of brain regions normally involved during a task and are observed when individuals are resting without performing a specific task (Biswal et al., 1995).

Research efforts in functional magnetic resonance imaging (fMRI) are shifting focus from studying specific cognitive domains like vision, language, memory, and emotion to assessing individual differences in neural connectivity across multiple whole-brain networks (Thomason et al., 2011). Subsequently, an increasing number of studies using rs-fMRI data, are showing reproducibility and reliability (Damoiseaux et al., 2006; Shehzad et al., 2009; Van Dijk et al., 2010; Zuo et al., 2010; Thomason et al., 2011; Song et al., 2012), for studying functional connectivity of the human brain.

Simultaneously, use of machine learning techniques for analyzing fMRI data has increased in popularity. In particular, Support Vector Machines (SVMs) have become widely used due to their ability to handle very high-dimensional data and their classification and prediction accuracy (Schölkopf and Smola, 2002; Ben-Hur and Weston, 2010; Meier et al., 2012). Various fMRI data analysis methods are currently used including seed-based analysis, independent component analysis (ICA), graph theory methods, but in this work we chose SVMs because they, unlike the others, offer the ability to classify and predict individual scans and output relevant features. A growing number of studies have shown that machine learning tools can be used to extract exciting new information from neuroimaging data (see Haynes and Rees, 2005; Norman et al., 2006; Cohen et al., 2011 for selective reviews).

With task-based fMRI data, LaConte et al. (2007) observed 80% classification accuracy of real-time brain state prediction using a linear kernel SVM on whole-brain, block-design, motor data and Poldrack et al. (2009) achieved 80% classification accuracy of predicting eight different cognitive tasks that an individual performed using a multi-class SVM (mcSVM) method.

Resting state fMRI data has been shown viable in classification and prediction. Craddock et al. (2009) used resting state functional connectivity MRI (rs-fcMRI) data to successfully distinguish between individuals with major depressive disorder from healthy controls with 95% accuracy using a linear classifier with a reliability filter for feature selection. Supekar et al. (2009) classified individuals as children or young-adults with 90% accuracy using a SVM classifier. Shen et al. (2010) achieved 81% accuracy for discrimination between schizophrenic patients and healthy controls using a SVM classifier and achieved 92% accuracy using a *C*-means clustering classifier with locally linear embedding (LLE) feature selection. Dosenbach et al. (2010), using a SVM method, achieved 91% accuracy for classification of individuals as either children or adults, and also predicted functional maturity for each participant’s brain using support vector machine regression (SVR).

One advantage of resting state data as opposed to task-based data is that the acquiring of resting data is not constrained by task difficulty and performance. This provides a potentially larger group of subjects that are not able to perform tasks (e.g., Alzheimer’s Disease patients, patients with severe stroke) on which studies can be done. There has been a great amount of progress made in describing typical and atypical brain activity at the group level with the use of fMRI, but, determining whether single fMRI scans contain enough information to classify and make predictions about individuals remains a critical challenge (Dosenbach et al., 2010). Our method builds on the classification and prediction of individual scans using multivariate pattern recognition algorithms, adding to this currently novel domain in the literature.

We describe a classification and regression method implemented on aging adult rs-fcMRI data using SVMs, extracting relevant features, and building on the SVM/SVR study of children to middle-aged subjects (Dosenbach et al., 2010) and aging adults (Meier et al., 2012). SVM has been applied to a wide range of datasets, but has only recently been applied to neuroimaging-fMRI data, especially resting fMRI data which is still relatively novel. This work expands upon and adds to the relatively new literature of resting fMRI based classification and prediction. Our objective was to investigate the ability of the SVM classifier to discriminate between individuals with respect to age and the ability of the SVR predictor to determine individuals’ age using only functional connectivity MRI data. Beyond binary SVM classification and SVR prediction, our work investigates multi-class classification and linear weights for evaluating feature importance of healthy aging adults.

## Materials and Methods

### Participants

Resting state data for 65 individuals (three scans each) were obtained from the ICBM dataset made freely accessible online by the 1000 Connectome Project^1^. Each contributor’s respective ethics committee approved submission of the de-identified data. The institutional review boards of NYU Langone Medical Center and New Jersey Medical School approved the receipt and dissemination of the data (Biswal et al., 2010).

### Data sets

The analyses described in this work were performed on two data sets contained in the ICBM set. The same preprocessing algorithms were applied to both sets of data.

Data set 1 consisted of 52 right-handed individuals (age 19–85, mean 44.7, 23M/29F). This was the binary SVM set (both for age and gender classification) which contained a young group of 26 subjects (age 19–35, mean 24.7, 12M/14F) and an old group of 26 subjects (age 55–85, mean 64.7, 11M/15F).

Data set 2 consisted of 65 right-handed individuals (ages 19–85, mean 44.9, 32M/33F). This was the mcSVM set as well as the SVR age prediction set. It contained three age groups used for mcSVM: a young group of 28 subjects (age 19–37, mean 25.5, 14M/14F), a middle-aged group of 22 subjects (age 42–60, mean 52.4, 11M/11F), and an old group of 15 subjects (age 61–85, mean 69.9, 7M/8F).

### Data acquisition

Resting data were acquired with a 3.0 Tesla scanner using an echo planar imaging (EPI) pulse sequence. Three resting state scans were obtained for each participant, and consisted of 128 continuous resting state volumes (TR = 2000 ms; matrix = 64 × 64; 23 axial slices). Scan 1 and 3 had an acquisition voxel size = 4 mm × 4 mm × 5.5 mm, while scan 2 had an acquisition voxel size = 4 mm × 4 mm × 4 mm. All participants were asked to keep their eyes closed during the scan. For spatial normalization and localization, a T1-weighted anatomical image was acquired using a magnetization prepared gradient echo sequence (MP-RAGE, 160 sagittal slices, voxel size = 1 mm × 1 mm × 1 mm).

### Data preprocessing

Data were preprocessed using AFNI (version AFNI_2009_12_31_1431^2^), FSL (version 4.1.4^3^), and the NITRC 1000 functional connectome preprocessing scripts made freely available online (version 1.1^4^) (Neuroimaging Informatics Tools and Resources Clearinghouse (NITRC), 2011). Initial preprocessing using AFNI consisted of (1) slice time correction for interleaved acquisition using Fourier-space time series phase-shifting, (2) motion correction of time series by aligning each volume to the mean image using Fourier interpolation, (3) skull stripping, and (4) getting an eighth image for use in registration. Preprocessing using FSL consisted of (5) spatial smoothing using a Gaussian kernel of full-width half maximum = 6 mm, and (6) grand-mean scaling of the voxel values. The data were then temporally filtered (0.005–0.1 Hz) and detrended to remove linear and quadratic trends using AFNI. A mask of preprocessed data for each person was generated.

### Nuisance signal regression

Nuisance signal [white matter, cerebrospinal fluid (CSF) and six motion parameters] was then removed from the preprocessed fMRI data. White matter and CSF masks were created using FSL by the segmentation of each individual’s structural image. These masks were then applied to each volume to remove the white matter and CSF signal. Following the removal of these nuisance signals, functional data were then transformed into Montreal Neurological Institute 152 (MNI152-brain template; voxel size = 3 mm × 3 mm × 3 mm) space using a two-step process. First a 6 degree-of-freedom affine transform was applied using FLIRT (Smith et al., 2004) to align the functional data into anatomical space. Then, the anatomical image was aligned into standard MNI space using a 12 degree-of-freedom affine transform implemented in FLIRT. Finally, the resulting transform was then applied to each subject’s functional dataset.

### ROI based functional connectivity

**Figure 1:**
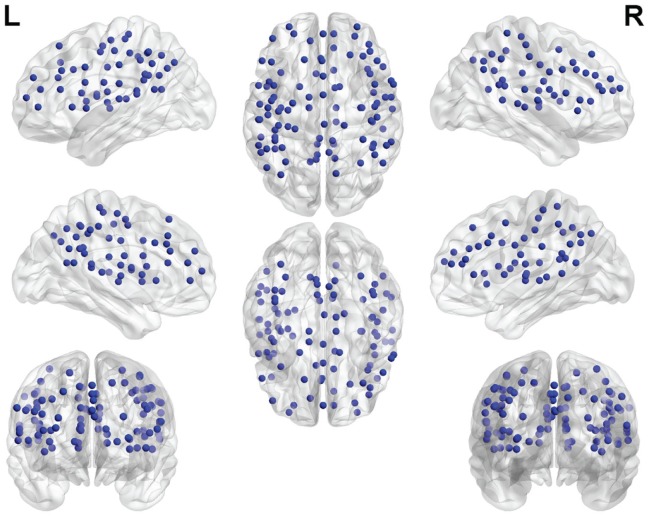
**Functional ROIs used in the study**. Each ROI is spherical with a 5 mm radius.

One hundred functionally defined regions of interest (ROIs) encompassing the default mode, cingulo-opercular, fronto-parietal, and sensorimotor networks (see Figure 1), were selected in agreement with a previous study by Dosenbach et al. (2010) and Meier et al. (2012). Each ROI was defined by a sphere (radius = 5 mm) centered about a three-dimensional point with coordinates reported in MNI space.

**Figure 2:**
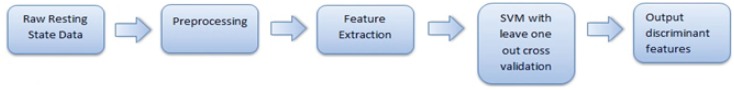
**Pipeline of the classification method**.

Average resting state blood oxygenation level dependent (BOLD) time series for each ROI were extracted. The BOLD time series for each ROI were then correlated with the BOLD time series of every other ROI (Pearson’s correlation) for every subject and every scan. This resulted in a square (100 × 100) symmetric matrix of correlation coefficients for each scan, but only 4950 ROI-pair correlation values from the lower triangular part of the matrix were retained (redundant elements and diagonal elements were excluded). These were then *z*-transformed (Fisher’s *z* transformation) for normalization. These 4950 values of the functional connectivity matrix were subsequently used as features in the SVM and SVR methods. Figure 2 shows a series of steps in a representative pipeline of the classification method.

### Support vector machine classification and regression

The SVM is a widely used classification method due to its favorable characteristics of high accuracy, ability to deal with high-dimensional data and versatility in modeling diverse sources of data (Schölkopf et al., 2004). We chose this method of classification due to its sensitivity, resilience to overfitting, ability to extract and interpret features, and recent history of impressive neuroimaging results (Mitchell et al., 2008; Soon et al., 2008; Johnson et al., 2009; Dosenbach et al., 2010; Schurger et al., 2010; Meier et al., 2012).

A SVM is an example of a linear two-class classifier, which is based on a linear discriminant function:

$$f\;(x_{i})=w\cdot x_{i}+b.$$

The vector **w** is the weight vector, *b* is called the bias and ***x***_i_ is the *i-th* example in the dataset. In our study we have a dataset of *n* examples each of *p* retained features, ***x***_i_ ∈ ℝ*^p^*, where *n* is the number of subjects and *p* is the number of retained ROI-pair correlation values after *t*-test filtering. Each example ***x***_i_ has a user defined label *y*_i_ = +1 or −1, corresponding to the class that it belongs to. In this work binary participant classes are young or old and male or female subjects.

A brief description of the SVM optimization problem is given here and a more detailed one can be found in Vapnik’s (1995) work and Schölkopf and Smola (2002). For linearly separable data, a hard margin SVM classifier is a discriminant function that maximizes the geometric margin, which leads to the following constrained optimization problem:

$$\begin{array}{c} \underset{w,b}{\min}\frac{1}{2}{\left\|w\right\|}^{2}\\ \text{subject to}:\;y_{i}(w\cdot x_{i}+b)\geq 1i=1,\ldots ,\;n. \end{array}$$

In the soft margin SVM (Cortes and Vapnik, 1995), where misclassification and non-linearly separable data are allowed, the problem constraints can be modified to:

$$y_{i}(w\cdot x_{i}+b)\geq 1-\xi_{i}\;i=1,\ldots ,n,$$

where ξ*_i_* ≥ 0 are slack variables that allow an example to be in the margin (0 ≤ ξ*_i_* ≤ 1), or to be misclassified (ξ*_i_* > 1). The optimization problem, with an additional term $C\;\sum_{i=1}^{n}\xi_{i}$ that penalizes misclassification and within margin examples, becomes:

$$\begin{array}{c} \underset{w,b}{\min}\frac{1}{2}{\left\|w\right\|}^{2}+C\sum_{i=1}^{n}\xi_{i}\\ \text{subject to}:\;y_{i}(w\cdot x_{i}+b)\geq 1-\xi_{i}\;i=1,\ldots ,n. \end{array}$$

The constant *C* > 0 allows one to control the relative importance of maximizing the margin and minimizing the amount of discriminating boundary and margin slack.

This can be represented in a dual formulation in terms of variables α_i_ (Cortes and Vapnik, 1995):

$$\begin{array}{l} \underset{\alpha }{\max}\;\sum_{i=1}^{n}\alpha_{i}-\frac{1}{2}\sum_{i=1}^{n}\sum_{j=1}^{n}y_{i}y_{j}\alpha_{i}\alpha_{j}x_{i}\cdot x_{j}\\ \text{ subject to: }\sum_{i=1}^{n}y_{i}\alpha_{i}=0,\;0\leq \alpha_{i}\leq C. \end{array}$$

The dual formulation leads to an expansion of the weight vector in terms of input data examples:

$$w=\sum_{i=1}^{n}y_{i}\alpha_{i}x_{i}.$$

The examples ***x***_i_ for which α_i_ > 0 are within the margin and are called support vectors.

The discriminant function then becomes:

$$f(x_{i})=\sum_{j=1}^{n}y_{i}\alpha_{i}x_{j}\cdot x_{i}+b.$$

The dual formulation of the optimization problem depends on the data only through dot products. This dot product can be replaced with a non-linear kernel function, *k*(***x****_i_*, ***x***_j_), enabling margin separation in the feature space of the kernel. Using a different kernel, in essence, maps the example points, ***x***_i_, into a new high-dimensional space (with the dimension not necessarily equal to the dimension of the original feature space). The discriminant function becomes:

$$f(x_{i})=\sum_{j=1}^{n}y_{i}\alpha_{i}k\left(x_{i},x_{j}\right)+b.$$

Some commonly used kernels are the polynomial kernel and the Gaussian kernel. In this work we used a linear kernel and a Gaussian kernel, which is also called a radial basis function (RBF):

$$\begin{array}{l} k\;\left(x_{i},x_{j}\right)=\text{exp }\left(-{\left\|x_{i}-x_{j}\right\|}^{2}/\left(2\sigma^{2}\right)\right),\\ \text{ with }\sigma =2. \end{array}$$

We tuned the value of *C* using a holdout subset of the respective dataset. Soft margin binary SVM classification was carried out using the Spider Machine Learning environment (Weston et al., 2005) as well as custom scripts run in MATLAB (R2010a; MathWorks, Natick, MA, USA). Multi-class classification was also carried out using the Spider Machine Learning environment (Weston et al., 2005) utilizing an algorithm, developed by Weston and Watkins (1998), that considers all data at once and solves a single optimization problem.

With some datasets higher classification accuracies can be obtained with the use of non-linear discriminating boundaries (Ben-Hur and Weston, 2010). Using a different kernel maps the data points into a new high-dimensional space, and in this space the SVM discriminating hyperplane is found. Consequently, in the original space, the discriminating boundary will not be linear. All SVM classification and SVR prediction in this work used a linear kernel or a non-linear RBF kernel.

Drucker et al. (1997) extended the SVM method to include SVM regression (SVR) in order to make continuous real-valued predictions. SVR retains some of the main features of SVM classification, but in SVM classification a penalty is observed for misclassified data points, whereas in SVR a penalty is observed for points too far from the regression line in high-dimensional space (Dosenbach et al., 2010).

Epsilon-insensitive SVR defines a tube of width ε, which is user defined, around the regression line in high-dimensional space. Any points within this tube carry no loss. In essence, SVR performs linear regression in high-dimensional space using epsilon-insensitive loss. The *C* parameter in SVR controls the trade-off between how strongly points beyond the epsilon-insensitive tube are penalized and the flatness of the regression line (larger values of *C* allow the regression line to be less flat) (Dosenbach et al., 2010). SVR predictions described in this work used epsilon-insensitive SVRs carried out in The Spider Machine Learning environment (Weston et al., 2005), as well as custom scripts run in MATLAB (R2010a; MathWorks, Natick, MA, USA). The parameters *C* and ε were tuned using a holdout subset of the respective dataset.

### Cross validation

We used leave-one-out-cross-validation (LOOCV) to estimate the SVM classification and SVR prediction accuracy since it is a method that gives the most unbiased estimate of test error (Hastie et al., 2001). In LOOCV the same dataset can be used for both the training and testing of the classifier. The SVM parameters: *C* and the number of top features, were tuned using a holdout set with LOOCV.

In a round, or fold, of LOOCV, an example from the example set is left out and is used as the entire testing set, while the remaining examples are used as the training set. So each example is left out only once and the number of folds is equal to the number of examples. In our work, LOOCV was performed across participants, not scans, so three scans per participant were removed in each fold and used only in the testing set to avoid “twinning” bias.

### *T*-test and correlation filter

During each SVM LOOCV fold, two-sample *t*-tests (not assuming equal variance) were run on every feature of the two classes of the training set and the number of features (selected to maximize accuracy) that had the highest absolute *t*-statistics were selected for use in the classifier. Analogously, during each SVR LOOCV fold, the correlation between each feature and the independent variable (age) was computed, and the features that had the highest absolute correlation values were selected for use in the predictor.

### SVM and SVR feature weights

One important aspect of SVM and SVR is the determination of which features in the model are most significant with respect to example classification and prediction.

For linear kernel SVM and SVR features, the individual weights of the features as given by the SVM or SVR revealed their relative importance and contribution to the classification or prediction. In the linear kernel SVM and SVR method each node’s (ROI’s) significance, as opposed to each feature’s significance, was directly proportional to the sum of the weights of the connections to and from that node.

### Feature and node visualization

Feature connections and nodes were visualized using BrainNet Viewer (Version 1.1^5^).

### Parameter tuning

Dosenbach et al. (2010) chose *C* = 1, top features = 200 for their SVM method and ε = 0.00001, top features = 200 for their SVR method since previous work on a subset of the data revealed that these values provided highest accuracy. Our functional connectivity features used 100 ROIs instead of 160 and this resulted in a different feature space than the one used in the aforementioned study. To tune our SVM parameters for our feature space, we selected a randomly chosen subset, a holdout set, of the respective dataset and chose parameters that maximized classification accuracy and prediction performance for this set.

**Figure 3:**
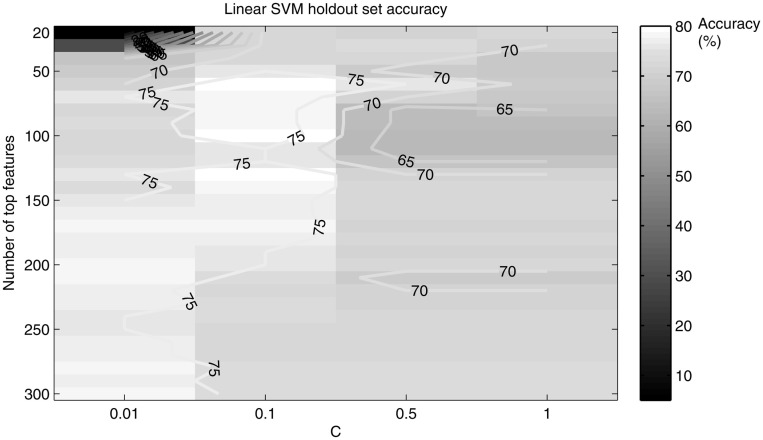
**A grid search plot of the hold out set linear SVM age classifier accuracy, as a function of the number of top features and *C***. Accuracy peaks at 80% for top features retained = 100 and *C* = 0.1.

A holdout set of 20 randomly chosen subjects was used to tune the SVM age and gender classification parameters. We limited ourselves to number of features <1000 for two reasons: previous work (Dosenbach et al., 2010) achieved highest accuracy for features on the order of 100, and this order provides a suitable number of features for characterizing the most relevant brain networks. A “grid search” like method (Hsu et al., 2010) was performed for an interval of number of top features ranging from 20 to 300 to output accuracy as a function of the number of top features and *C* (see Figure 3). The number of features and value of *C* that maximized accuracy were used in the total dataset SVM method.

A similar procedure for the SVR method was taken. A holdout set of 25 randomly chosen subjects was used to tune the SVR age prediction parameters. First, slope (of a linear regression line fitting the predicted age) as a function of top features was computed to reveal a peak performance area. Then, slope as a function of the number of features and ε was output with a grid search method. The number of features and value of ε that maximized the slope and *R*^2^ were used in the total dataset SVR method, where *R*^2^ (in this simple linear regression model) is the squared correlation between the predicted and true age. The slope and *R*^2^ of a regression line were chosen as measures of performance since a perfect predictor would produce a regression line of ŷ=1x+0; the closer the slope and *R*^2^ approached one the better the predictor was considered to be.

## Results

### Support vector machine

The binary SVM classifier, using a linear kernel, was able to significantly discriminate between young and old subjects with 84% accuracy (*p*-value < 1 × 10^−7^, binomial test). Chance performance of the classifier would have yielded an accuracy of 50% (the null hypothesis). Therefore, we treated each fold of the LOOCV as a Bernoulli trial with a success probability of 0.5, as specified by Pereira et al. (2009). The *p*-value is then calculated using the binomial distribution with *n* trials (*n* = number of subjects) and probability of success equal to 0.5 as follows: *p*-value = Pr(*X* ≥ number of correct classifications), where *X* is the binomially distributed random variable.

The linear kernel SVM classifier outperformed the RBF kernel SVM classifier with this dataset and a comparison of the two classifiers is given in Table 1. Figure 3 shows how the linear SVM classification accuracy varied with the number of top features retained in the *t*-test filter as well as how the accuracy varied as a function of the *C* parameter. The RBF SVM accuracy was 81% with 62 top features retained and *C* = 1.

**Table 1:** **A comparison of the two kernel classifiers used for age classification**.

Classifier	Accuracy (%)	Top features retained	*C*
Linear SVM	84	100	0.1
Rbf SVM	81	62	1.0

*Listed are the accuracy, number of top features retained and the value of *C* for each classifier*.

**Figure 4:**
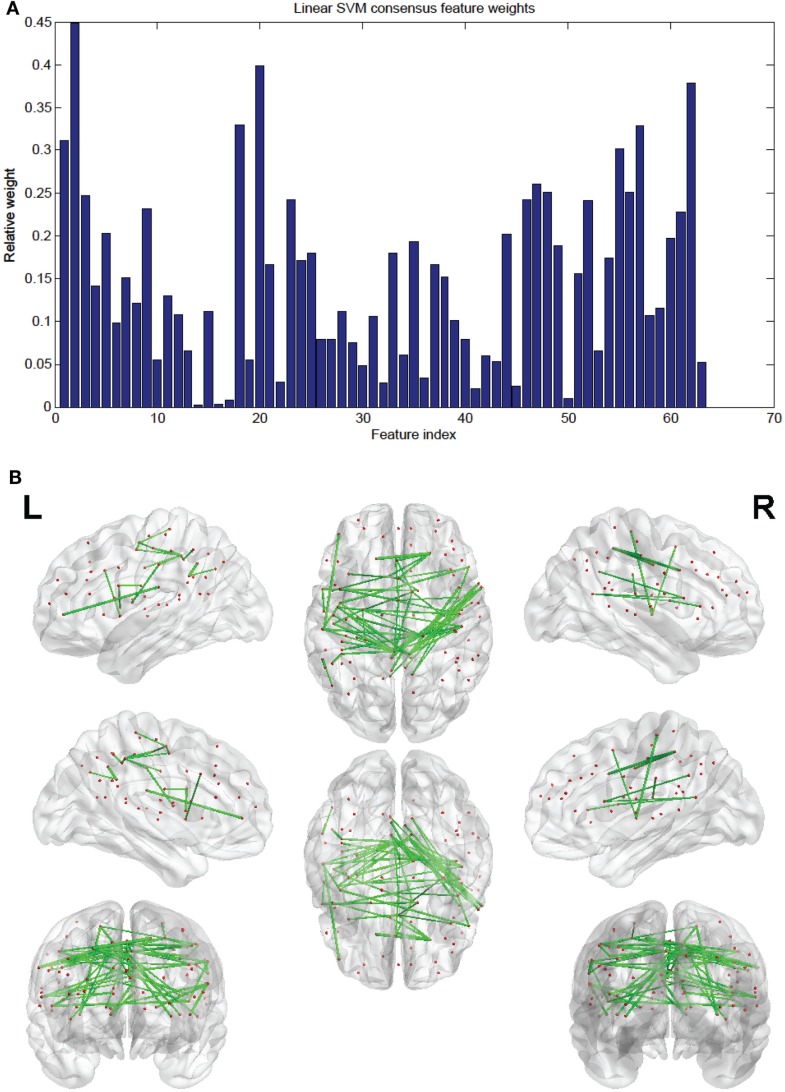
**(A)** Shows a bar graph representation of the relative weight of each of the 63 consensus features. **(B)** Shows a representation of the consensus features revealing location using BrainNet Viewer software. Each connection thickness is proportional to the feature weight.

**Figure 5:**
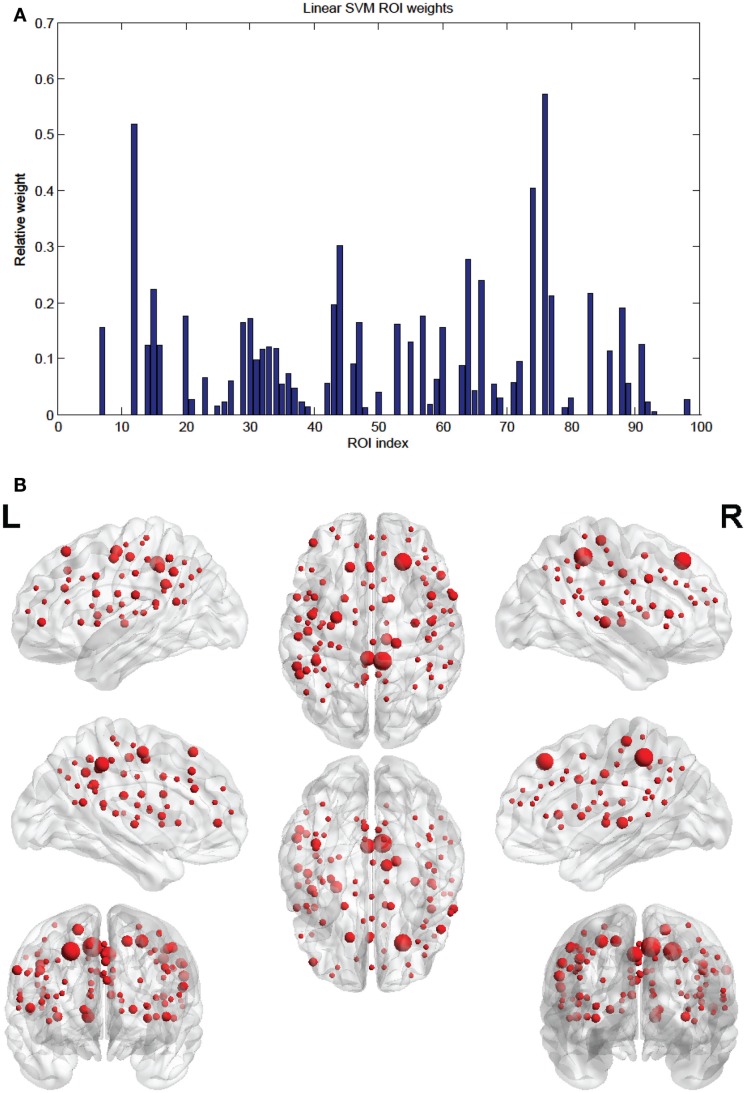
**(A)** Shows a bar graph representation of the relative weight or contribution of each node to the classifier. **(B)** Shows a representation of the weighted nodes revealing location using BrainNet Viewer software. Each node’s size is proportional to its weight.

Of the 100 total features retained per fold, 63 were present in every fold and these are called the consensus features. Table 2 lists the consensus features and their relative weights or contributions to the classifier; they are also represented in Figure 4. A summation of all of the weights of the connections from each node was performed and the node weights are listed in Table 3 and represented in Figure 5.

**Table 2:** **List of the 63 consensus features, their node connections and weights for the linear SVM classifier**.

Feature index	SVM feature number	ROI 1	Connected with	ROI 2	Weight
1	632	L_precentral_gyrus_3		L_vent_aPFC	0.3119
2	1037	L_sup_frontal		R_sup_frontal	0.4479
3	1038	M_ACC_2		R_sup_frontal	0.2472
4	1047	L_basal_ganglia_1		R_sup_frontal	0.1405
5	1048	M_mFC		R_sup_frontal	0.203
6	1231	R_pre_SMA		M_ACC_1	0.0986
7	1233	M_SMA		M_ACC_1	0.1508
8	1727	R_vFC_2		R_vFC_1	0.121
9	1732	L_mid_insula_1		R_vFC_1	0.2313
10	1795	M_mFC		R_ant_insula	0.0542
11	1950	M_mFC		L_ant_insula	0.1294
12	2110	L_vFC_3		L_basal_ganglia_1	0.1074
13	2183	R_basal_ganglia_1		M_mFC	0.0652
14	2301	L_post_cingulate_1		R_frontal_1	0.0016
15	2311	R_precuneus_3		R_frontal_1	0.1118
16	2314	R_post_cingulate		R_frontal_1	0.0027
17	2315	L_precuneus_2		R_frontal_1	0.0074
18	2441	R_precuneus_1		R_dFC_2	0.3302
19	2509	L_precuneus_1		R_dFC_3	0.0548
20	2511	R_precuneus_1		R_dFC_3	0.3977
21	2542	M_SMA		L_dFC	0.1668
22	2551	R_precentral_gyrus_3		L_dFC	0.029
23	2605	L_basal_ganglia_2		L_vFC_2	0.2421
24	2606	R_basal_ganglia_1		L_vFC_2	0.1719
25	2618	L_precentral_gyrus_2		L_vFC_2	0.1803
26	2884	L_mid_insula_2		R_pre_SMA	0.0787
27	2887	R_mid_insula_2		R_pre_SMA	0.0787
28	2908	L_precuneus_1		R_pre_SMA	0.112
29	2935	M_SMA		R_vFC_2	0.0752
30	2989	R_post_cingulate		R_vFC_2	0.0487
31	3033	L_precuneus_1		M_SMA	0.1055
32	3094	L_precuneus_1		R_frontal_2	0.0269
33	3256	L_parietal_5		L_mid_insula_1	0.1804
34	3277	R_precuneus_2		L_mid_insula_1	0.0604
35	3298	L_parietal_1		L_precentral_gyrus_1	0.1927
36	3328	L_precuneus_1		L_precentral_gyrus_1	0.0331
37	3330	R_precuneus_1		L_precentral_gyrus_1	0.1669
38	3357	R_precentral_gyrus_3		L_parietal_1	0.1524
39	3367	L_parietal_4		L_parietal_1	0.1008
40	3368	R_parietal_1		L_parietal_1	0.0787
41	3376	R_parietal_3		L_parietal_1	0.021
42	3379	L_parietal_7		L_parietal_1	0.0593
43	3546	L_precuneus_1		R_precentral_gyrus_3	0.0535
44	3548	R_precuneus_1		R_precentral_gyrus_3	0.2019
45	3598	L_precuneus_1		L_parietal_2	0.0234
46	3835	R_parietal_3		R_mid_insula_2	0.2415
47	3926	R_parietal_3		L_mid_insula_3	0.2598
48	4021	L_precuneus_1		L_parietal_4	0.2507
49	4061	L_temporal_2		R_parietal_1	0.1886
50	4063	L_precuneus_1		R_parietal_1	0.0089
51	4065	R_precuneus_1		R_parietal_1	0.1549
52	4095	M_post_cingulate		L_parietal_5	0.241
53	4104	L_precuneus_1		L_parietal_5	0.0656
54	4249	M_post_cingulate		R_post_insula	0.1736
55	4299	L_post_cingulate_1		R_basal_ganglia_2	0.3015
56	4311	L_post_cingulate_2		R_basal_ganglia_2	0.2509
57	4334	L_post_cingulate_1		M_post_cingulate	0.3287
58	4430	R_precuneus_1		L_post_insula	0.1071
59	4518	L_precuneus_1		L_post_parietal_1	0.1153
60	4602	L_IPL_1		L_precuneus_1	0.1964
61	4683	L_IPL_2		L_IPL_1	0.2273
62	4802	L_IPL_3		L_parietal_8	0.379
63	4812	L_angular_gyrus_2		L_parietal_8	0.0522

**Table 3:** **Linear SVM nodes and their weights**.

ROI index	ROI	Weight
7	L_vent_aPFC	0.1559
12	R_sup_frontal	0.5193
14	M_ACC_1	0.1247
15	L_sup_frontal	0.2239
16	M_ACC_2	0.1236
20	R_vFC_1	0.1761
21	R_ant_insula	0.0271
23	L_ant_insula	0.0647
25	L_basal_ganglia_1	0.0165
26	M_mFC	0.0229
27	R_frontal_1	0.0591
29	R_dFC_2	0.1651
30	R_dFC_3	0.1714
31	L_dFC	0.0979
32	L_vFC_2	0.1169
33	L_basal_ganglia_2	0.1211
34	R_basal_ganglia_1	0.1185
35	L_vFC_3	0.0537
36	R_pre_SMA	0.072
37	R_vFC_2	0.0473
38	M_SMA	0.0232
39	R_frontal_2	0.0135
42	L_mid_insula_1	0.0556
43	L_precentral_gyrus_1	0.1963
44	L_parietal_1	0.3025
46	L_precentral_gyrus_2	0.0901
47	R_precentral_gyrus_3	0.1649
48	L_parietal_2	0.0117
50	L_mid_insula_2	0.0394
53	R_mid_insula_2	0.1601
55	L_mid_insula_3	0.1299
57	L_parietal_4	0.1758
58	R_parietal_1	0.0181
59	L_parietal_5	0.0631
60	L_precentral_gyrus_3	0.1559
63	R_post_insula	0.0868
64	R_basal_ganglia_2	0.2762
65	M_post_cingulate	0.043
66	R_parietal_3	0.2401
68	L_post_insula	0.0536
69	L_parietal_7	0.0296
71	L_post_parietal_1	0.0577
72	L_temporal_2	0.0943
74	L_precuneus_1	0.4059
76	R_precuneus_1	0.5722
77	L_IPL_1	0.2118
79	L_post_cingulate_1	0.0128
80	R_precuneus_2	0.0302
83	L_parietal_8	0.2156
86	L_IPL_2	0.1137
88	L_IPL_3	0.1895
89	R_precuneus_3	0.0559
91	L_post_cingulate_2	0.1255
92	R_post_cingulate	0.023
93	L_precuneus_2	0.0037
98	L_angular_gyrus_2	0.0261

*Omitted nodes have a weight of zero*.

We employed the same SVM method on gender classification as we did for age classification. A linear SVM classifier was not able to significantly discriminate between male and female subjects (55% accuracy, *p*-value < 0.17, binomial test; compared to 50% for random chance). Also a multi-class linear kernel SVM classifier was applied to 65 subjects partitioned into three age groups: young, middle, and old. It was able to significantly discriminate between the three groups using a linear SVM with 28 top features retained and *C* = 0.1 (57% accuracy; *p*-value < 1 × 10^−4^, binomial test; compared to ∼33% for random chance).

### SVR

**Figure 6:**
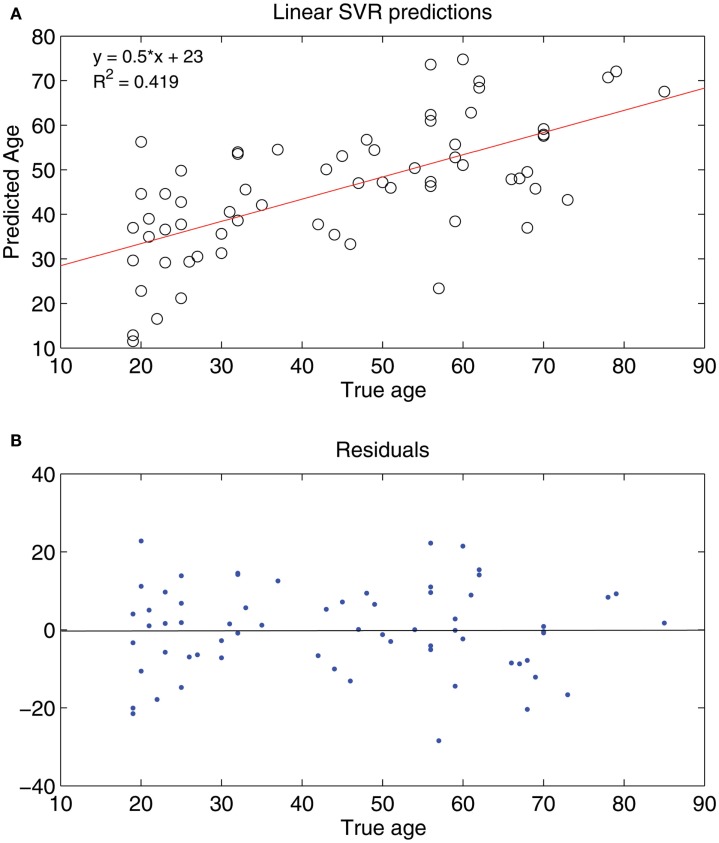
**(A)** Shows a least squares regression line on the predicted and actual age points. **(B)** Shows the residuals for the least squares regression fit.

**Figure 7:**
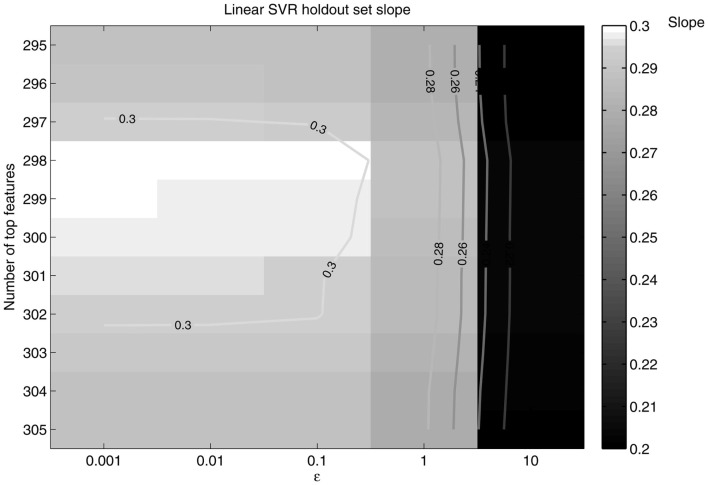
**Slope as a function of ε and the number of top features retained**. The slope peaks at 298 features retained and ε = 0.1.

**Figure 8:**
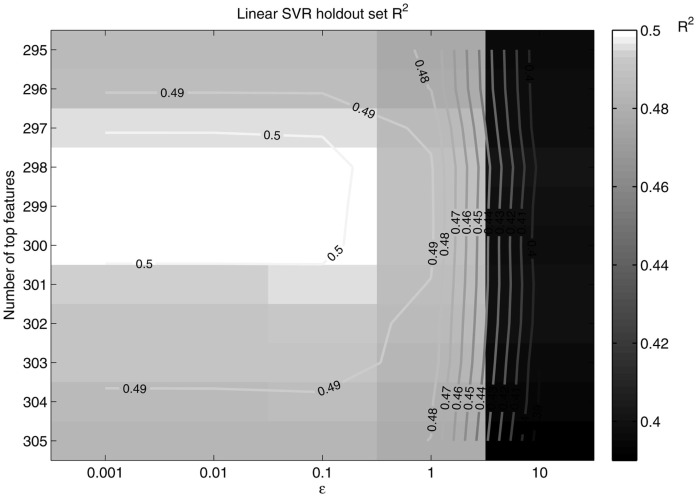
***R*^2^ as a function of ε and the number of top features retained**. *R*^2^ peaks at around 298 features retained and ε = 0.1, in the same neighborhood as the peak slope.

Seeing that classification of age groups was successful, we decided to test whether age prediction of individuals is viable on a continuous scale with the use of only fcMRI data. That is, given an fMRI connectivity map, we wanted to determine the age in years of the individual on a continuous range rather than choose between two or three discrete classes. A SVR linear predictor (top features retained = 298, ε = 0.1) was applied to 65 subjects varying in age (19–85 years) and was able to predict subject age with a reasonable degree of accuracy, [$\hat{y}=0.5x+23,\;R^{2}=0.419$, *p*-value < 1 × 10^−8^ (null hypothesis of no correlation or a slope of zero)], where $\hat{y}$ is a linear regression line applied to the (*x*, *y*) points with *x* being the true age of the subject and *y* the predicted age (see Figure 6). A similar holdout set method was employed for the SVR predictor as was for the SVM classifiers (see Figures 7 and 8).

**Figure 9:**
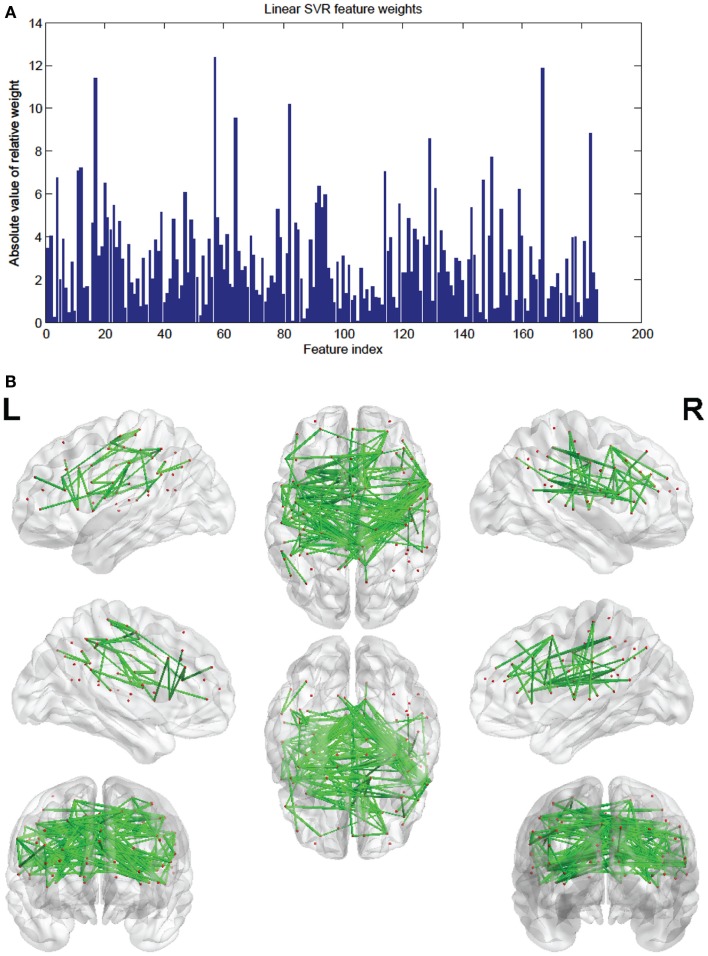
**(A)** Shows a bar graph representation of the relative weight or contribution of each of the 185 consensus features to the linear kernel SVR predictor. **(B)** Shows a representation of the 185 consensus features revealing location. Each connection thickness is proportional to the feature weight.

**Figure 10:**
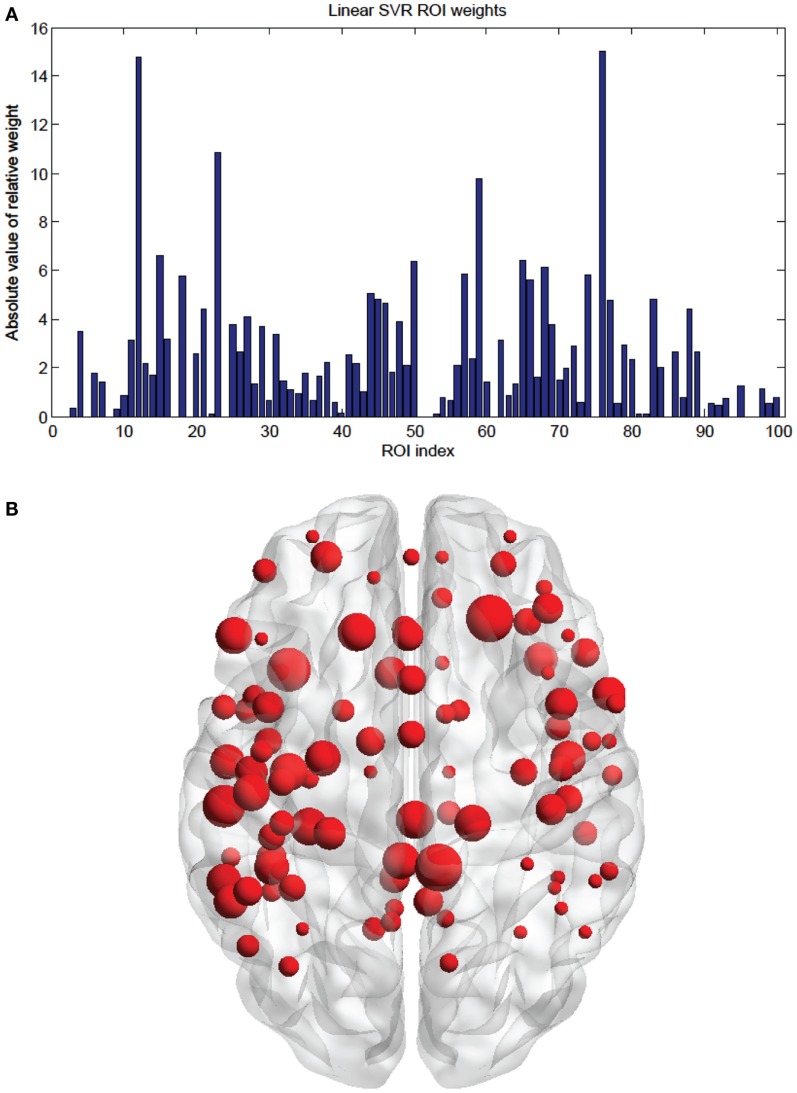
**(A)** Shows a bar graph representation of the relative weight or contribution of each node to the linear kernel SVR predictor, with ε fixed at 0.1. **(B)** Shows a representation of the 100 weighted nodes revealing location. Each node’s size is proportional to its weight.

The SVR method had 185 features (out of the 298) present in every fold. These consensus features’ weights and the node weights were computed in the same way as for the SVM classifier (see Figures 9 and 10; Tables 4 and 5).

**Table 4:** **A list of the consensus features and their weights for the linear SVR age predictor**.

Feature index	SVR feature number		ROI 1	Connected with	ROI 2	Weight
1	200	6	R_aPFC_2	3	M_mPFC	3.4199
2	208	14	M_ACC_1	3	M_mPFC	4.0323
3	302	12	R_sup_frontal	4	L_aPFC_2	0.1837
4	308	18	L_vPFC	4	L_aPFC_2	6.7691
5	514	35	L_vFC_3	6	R_aPFC_2	1.9686
6	515	36	R_pre_SMA	6	R_aPFC_2	3.9004
7	517	38	M_SMA	6	R_aPFC_2	1.5754
8	523	44	L_parietal_1	6	R_aPFC_2	0.4170
9	632	60	L_precentral_gyrus_3	7	L_vent_aPFC	2.8031
10	785	30	R_dFC_3	9	R_vlPFC	0.5353
11	871	26	M_mFC	10	R_ACC	7.0751
12	881	36	R_pre_SMA	10	R_ACC	7.2001
13	910	65	M_post_cingulate	10	R_ACC	1.5674
14	955	21	R_ant_insula	11	R_dlPFC_1	1.6761
15	957	23	L_ant_insula	11	R_dlPFC_1	0.0260
16	961	27	R_frontal_1	11	R_dlPFC_1	4.6158
17	1037	15	L_sup_frontal	12	R_sup_frontal	11.408
18	1038	16	M_ACC_2	12	R_sup_frontal	3.0458
19	1044	22	R_dACC	12	R_sup_frontal	3.5010
20	1047	25	L_basal_ganglia_1	12	R_sup_frontal	6.4695
21	1048	26	M_mFC	12	R_sup_frontal	4.8864
22	1139	30	R_dFC_3	13	R_vPFC	4.3102
23	1213	18	L_vPFC	14	M_ACC_1	5.4143
24	1218	23	L_ant_insula	14	M_ACC_1	3.4732
25	1231	36	R_pre_SMA	14	M_ACC_1	4.6873
26	1233	38	M_SMA	14	M_ACC_1	2.9394
27	1239	44	L_parietal_1	14	M_ACC_1	0.6638
28	1260	65	M_post_cingulate	14	M_ACC_1	3.6222
29	1306	26	M_mFC	15	L_sup_frontal	1.8433
30	1387	23	L_ant_insula	16	M_ACC_2	1.2731
31	1398	34	R_basal_ganglia_1	16	M_ACC_2	2.0425
32	1560	31	L_dFC	18	L_vPFC	0.7004
33	1727	37	R_vFC_2	20	R_vFC_1	2.9906
34	1730	40	R_precentral_gyrus_1	20	R_vFC_1	0.8001
35	1732	42	L_mid_insula_1	20	R_vFC_1	3.3381
36	1739	49	R_mid_insula_1	20	R_vFC_1	2.0195
37	1791	22	R_dACC	21	R_ant_insula	3.8335
38	1795	26	M_mFC	21	R_ant_insula	3.2795
39	1870	23	L_ant_insula	22	R_dACC	5.1255
40	1880	33	L_basal_ganglia_2	22	R_dACC	0.8944
41	1881	34	R_basal_ganglia_1	22	R_dACC	1.3206
42	1882	35	L_vFC_3	22	R_dACC	2.0233
43	1883	36	R_pre_SMA	22	R_dACC	4.7918
44	1949	25	L_basal_ganglia_1	23	L_ant_insula	2.8737
45	1950	26	M_mFC	23	L_ant_insula	1.0806
46	1954	30	R_dFC_3	23	L_ant_insula	1.6872
47	1960	36	R_pre_SMA	23	L_ant_insula	6.0690
48	2006	82	R_IPL_1	23	L_ant_insula	2.2798
49	2016	92	R_post_cingulate	23	L_ant_insula	4.7532
50	2110	35	L_vFC_3	25	L_basal_ganglia_1	3.9079
51	2113	38	M_SMA	25	L_basal_ganglia_1	2.0904
52	2176	27	R_frontal_1	26	M_mFC	0.2893
53	2182	33	L_basal_ganglia_2	26	M_mFC	3.0670
54	2183	34	R_basal_ganglia_1	26	M_mFC	0.8171
55	2184	35	L_vFC_3	26	M_mFC	3.9006
56	2190	41	L_thalamus_1	26	M_mFC	2.0477
57	2217	68	L_post_insula	26	M_mFC	12.328
58	2252	30	R_dFC_3	27	R_frontal_1	4.8758
59	2258	36	R_pre_SMA	27	R_frontal_1	3.5564
60	2262	40	R_precentral_gyrus_1	27	R_frontal_1	2.4206
61	2267	45	R_precentral_gyrus_2	27	R_frontal_1	4.0491
62	2271	49	R_mid_insula_1	27	R_frontal_1	1.7481
63	2299	77	L_IPL_1	27	R_frontal_1	1.6080
64	2301	79	L_post_cingulate_1	27	R_frontal_1	9.5243
65	2302	80	R_precuneus_2	27	R_frontal_1	3.2888
66	2304	82	R_IPL_1	27	R_frontal_1	2.3834
67	2308	86	L_IPL_2	27	R_frontal_1	2.5869
68	2311	89	R_precuneus_3	27	R_frontal_1	1.6131
69	2313	91	L_post_cingulate_2	27	R_frontal_1	4.0015
70	2314	92	R_post_cingulate	27	R_frontal_1	3.1354
71	2315	93	L_precuneus_2	27	R_frontal_1	1.4905
72	2317	95	L_post_cingulate_3	27	R_frontal_1	1.2658
73	2340	46	L_precentral_gyrus_2	28	L_vFC_1	2.9832
74	2343	49	R_mid_insula_1	28	L_vFC_1	0.9104
75	2344	50	L_mid_insula_2	28	L_vFC_1	1.5884
76	2374	80	R_precuneus_2	28	L_vFC_1	2.1640
77	2399	34	R_basal_ganglia_1	29	R_dFC_2	1.8317
78	2439	74	L_precuneus_1	29	R_dFC_2	5.2682
79	2441	76	R_precuneus_1	29	R_dFC_2	3.9293
80	2472	37	R_vFC_2	30	R_dFC_3	1.2744
81	2509	74	L_precuneus_1	30	R_dFC_3	3.1864
82	2511	76	R_precuneus_1	30	R_dFC_3	10.158
83	2540	36	R_pre_SMA	31	L_dFC	0.0347
84	2542	38	M_SMA	31	L_dFC	4.5939
85	2551	47	R_precentral_gyrus_3	31	L_dFC	4.2930
86	2561	57	L_parietal_4	31	L_dFC	2.0379
87	2562	58	R_parietal_1	31	L_dFC	0.1465
88	2570	66	R_parietal_3	31	L_dFC	0.6321
89	2573	69	L_parietal_7	31	L_dFC	3.8353
90	2606	34	R_basal_ganglia_1	32	L_vFC_2	1.6084
91	2617	45	R_precentral_gyrus_2	32	L_vFC_2	5.5595
92	2618	46	L_precentral_gyrus_2	32	L_vFC_2	6.3606
93	2620	48	L_parietal_2	32	L_vFC_2	5.3524
94	2806	36	R_pre_SMA	35	L_vFC_3	5.9420
95	2829	59	L_parietal_5	35	L_vFC_3	2.5231
96	2876	42	L_mid_insula_1	36	R_pre_SMA	2.0429
97	2884	50	L_mid_insula_2	36	R_pre_SMA	0.8906
98	2887	53	R_mid_insula_2	36	R_pre_SMA	2.8127
99	2889	55	L_mid_insula_3	36	R_pre_SMA	0.6196
100	2908	74	L_precuneus_1	36	R_pre_SMA	3.0879
101	2935	38	M_SMA	37	R_vFC_2	1.3538
102	2977	80	R_precuneus_2	37	R_vFC_2	2.6368
103	2989	92	R_post_cingulate	37	R_vFC_2	1.0198
104	2992	95	L_post_cingulate_3	37	R_vFC_2	1.2270
105	3001	42	L_mid_insula_1	38	M_SMA	0.0421
106	3009	50	L_mid_insula_2	38	M_SMA	2.5175
107	3012	53	R_mid_insula_2	38	M_SMA	1.0882
108	3013	54	R_temporal_1	38	M_SMA	1.5278
109	3022	63	R_post_insula	38	M_SMA	0.5381
110	3033	74	L_precuneus_1	38	M_SMA	1.6784
111	3094	74	L_precuneus_1	39	R_frontal_2	1.1764
112	3160	80	R_precuneus_2	40	R_precentral_gyrus_1	1.1140
113	3172	92	R_post_cingulate	40	R_precentral_gyrus_1	0.7809
114	3181	42	L_mid_insula_1	41	L_thalamus_1	7.0436
115	3255	58	R_parietal_1	42	L_mid_insula_1	3.3164
116	3256	59	L_parietal_5	42	L_mid_insula_1	3.9536
117	3268	71	L_post_parietal_1	42	L_mid_insula_1	1.1748
118	3274	77	L_IPL_1	42	L_mid_insula_1	0.6810
119	3276	79	L_post_cingulate_1	42	L_mid_insula_1	5.5190
120	3277	80	R_precuneus_2	42	L_mid_insula_1	2.2915
121	3289	92	R_post_cingulate	42	L_mid_insula_1	2.2737
122	3298	44	L_parietal_1	43	L_precentral_gyrus_1	4.8264
123	3320	66	R_parietal_3	43	L_precentral_gyrus_1	2.3284
124	3328	74	L_precuneus_1	43	L_precentral_gyrus_1	4.3556
125	3330	76	R_precuneus_1	43	L_precentral_gyrus_1	3.8301
126	3357	47	R_precentral_gyrus_3	44	L_parietal_1	1.4419
127	3363	53	R_mid_insula_2	44	L_parietal_1	3.9555
128	3366	56	L_parietal_3	44	L_parietal_1	3.5900
129	3367	57	L_parietal_4	44	L_parietal_1	8.5639
130	3368	58	R_parietal_1	44	L_parietal_1	0.9669
131	3372	62	R_parietal_2	44	L_parietal_1	6.2692
132	3376	66	R_parietal_3	44	L_parietal_1	2.2942
133	3377	67	L_parietal_6	44	L_parietal_1	4.2380
134	3379	69	L_parietal_7	44	L_parietal_1	3.3560
135	3386	76	R_precuneus_1	44	L_parietal_1	2.3440
136	3521	49	R_mid_insula_1	47	R_precentral_gyrus_3	1.7159
137	3537	65	M_post_cingulate	47	R_precentral_gyrus_3	1.2226
138	3542	70	R_temporal_2	47	R_precentral_gyrus_3	2.9690
139	3546	74	L_precuneus_1	47	R_precentral_gyrus_3	2.8436
140	3548	76	R_precuneus_1	47	R_precentral_gyrus_3	1.9436
141	3553	81	R_temporal_3	47	R_precentral_gyrus_3	0.1955
142	3598	74	L_precuneus_1	48	L_parietal_2	2.8843
143	3600	76	R_precuneus_1	48	L_parietal_2	5.3379
144	3633	58	R_parietal_1	49	R_mid_insula_1	3.0962
145	3634	59	L_parietal_5	49	R_mid_insula_1	1.2744
146	3683	58	R_parietal_1	50	L_mid_insula_2	0.4134
147	3684	59	L_parietal_5	50	L_mid_insula_2	6.6085
148	3690	65	M_post_cingulate	50	L_mid_insula_2	0.1353
149	3705	80	R_precuneus_2	50	L_mid_insula_2	4.0167
150	3835	66	R_parietal_3	53	R_mid_insula_2	7.7145
151	3926	66	R_parietal_3	55	L_mid_insula_3	0.6183
152	3973	69	L_parietal_7	56	L_parietal_3	0.6484
153	4021	74	L_precuneus_1	57	L_parietal_4	5.2334
154	4061	72	L_temporal_2	58	R_parietal_1	2.2855
155	4062	73	L_temporal_3	58	R_parietal_1	1.2126
156	4063	74	L_precuneus_1	58	R_parietal_1	3.3874
157	4065	76	R_precuneus_1	58	R_parietal_1	0.0311
158	4095	65	M_post_cingulate	59	L_parietal_5	1.0257
159	4104	74	L_precuneus_1	59	L_parietal_5	6.1957
160	4249	65	M_post_cingulate	63	R_post_insula	4.0141
161	4253	69	L_parietal_7	63	R_post_insula	1.0645
162	4255	71	L_post_parietal_1	63	R_post_insula	0.5269
163	4264	80	R_precuneus_2	63	R_post_insula	3.5363
164	4286	66	R_parietal_3	64	R_basal_ganglia_2	2.2027
165	4299	79	L_post_cingulate_1	64	R_basal_ganglia_2	1.9985
166	4311	91	L_post_cingulate_2	64	R_basal_ganglia_2	2.8954
167	4334	79	L_post_cingulate_1	65	M_post_cingulate	11.855
168	4335	80	R_precuneus_2	65	M_post_cingulate	0.2271
169	4400	78	R_parietal_4	67	L_parietal_6	1.0522
170	4430	76	R_precuneus_1	68	L_post_insula	1.6719
171	4441	87	L_angular_gyrus_1	68	L_post_insula	1.6202
172	4516	72	L_temporal_2	71	L_post_parietal_1	2.2346
173	4518	74	L_precuneus_1	71	L_post_parietal_1	1.3602
174	4521	77	L_IPL_1	71	L_post_parietal_1	0.1952
175	4530	86	L_IPL_2	71	L_post_parietal_1	2.9293
176	4552	80	R_precuneus_2	72	L_temporal_2	1.2155
177	4602	77	L_IPL_1	74	L_precuneus_1	3.9390
178	4609	84	L_post_parietal_2	74	L_precuneus_1	3.9741
179	4651	77	L_IPL_1	76	R_precuneus_1	0.8857
180	4683	86	L_IPL_2	77	L_IPL_1	0.2140
181	4686	89	R_precuneus_3	77	L_IPL_1	3.7542
182	4759	99	R_precuneus_4	80	R_precuneus_2	1.0681
183	4802	88	L_IPL_3	83	L_parietal_8	8.7927
184	4812	98	L_angular_gyrus_2	83	L_parietal_8	2.3099
185	4814	100	L_IPS_2	83	L_parietal_8	1.5265

**Table 5:** **Nodes and their weights for the linear kernel SVR predictor**.

ROI index	ROI	Weight
3	M_mPFC	0.3062
4	L_aPFC_2	3.4764
6	R_aPFC_2	1.7403
7	L_vent_aPFC	1.4016
9	R_vlPFC	0.2676
10	R_ACC	0.8462
11	R_dlPFC_1	3.1330
12	R_sup_frontal	14.747
13	R_vPFC	2.1551
14	M_ACC_1	1.7177
15	L_sup_frontal	6.6258
16	M_ACC_2	3.1807
18	L_vPFC	5.7415
20	R_vFC_1	2.5547
21	R_ant_insula	4.3946
22	R_dACC	0.0952
23	L_ant_insula	10.848
25	L_basal_ganglia_1	3.7628
26	M_mFC	2.6519
27	R_frontal_1	4.1057
28	L_vFC_1	1.3242
29	R_dFC_2	3.6829
30	R_dFC_3	0.6411
31	L_dFC	3.3619
32	L_vFC_2	1.4715
33	L_basal_ganglia_2	1.0863
34	R_basal_ganglia_1	0.9506
35	L_vFC_3	1.7332
36	R_pre_SMA	0.6284
37	R_vFC_2	1.6506
38	M_SMA	2.2000
39	R_frontal_2	0.5882
40	R_precentral_gyrus_1	0.1372
41	L_thalamus_1	2.4979
42	L_mid_insula_1	2.1402
43	L_precentral_gyrus_1	0.9863
44	L_parietal_1	5.0775
45	R_precentral_gyrus_2	4.8043
46	L_precentral_gyrus_2	4.6719
47	R_precentral_gyrus_3	1.8094
48	L_parietal_2	3.9030
49	R_mid_insula_1	2.0883
50	L_mid_insula_2	6.3615
53	R_mid_insula_2	0.0710
54	R_temporal_1	0.7639
55	L_mid_insula_3	0.6189
56	L_parietal_3	2.1192
57	L_parietal_4	5.8797
58	R_parietal_1	2.3834
59	L_parietal_5	9.7648
60	L_precentral_gyrus_3	1.4016
62	R_parietal_2	3.1346
63	R_post_insula	0.8258
64	R_basal_ganglia_2	1.3456
65	M_post_cingulate	6.4323
66	R_parietal_3	5.6008
67	L_parietal_6	1.5929
68	L_post_insula	6.1384
69	L_parietal_7	3.8037
70	R_temporal_2	1.4845
71	L_post_parietal_1	1.9759
72	L_temporal_2	2.8678
73	L_temporal_3	0.6063
74	L_precuneus_1	5.8246
76	R_precuneus_1	15.035
77	L_IPL_1	4.7624
78	R_parietal_4	0.5261
79	L_post_cingulate_1	2.9254
80	R_precuneus_2	2.2972
81	R_temporal_3	0.0978
82	R_IPL_1	0.0518
83	L_parietal_8	4.7881
84	L_post_parietal_2	1.9870
86	L_IPL_2	2.6511
87	L_angular_gyrus_1	0.8101
88	L_IPL_3	4.3964
89	R_precuneus_3	2.6837
91	L_post_cingulate_2	0.5530
92	R_post_cingulate	0.4474
93	L_precuneus_2	0.7453
95	L_post_cingulate_3	1.2464
98	L_angular_gyrus_2	1.1549
99	R_precuneus_4	0.5340
100	L_IPS_2	0.7632

*Omitted nodes have a weight of zero*.

**Figure 11:**
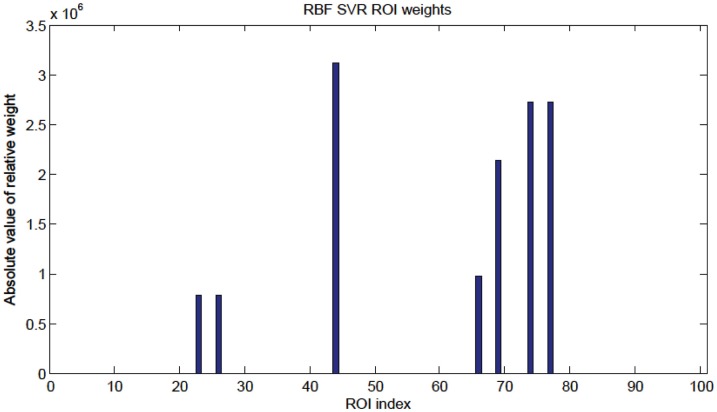
**Radial basis function kernel SVR node weights**. Since the RBF SVR method used 15 top features total, only seven nodes were present as shown in Table 6.

To check for agreement with previous studies (see Dosenbach et al., 2010), a SVR predictor using a RBF kernel was applied to our same 65 subject data set. The RBF SVR predictor (top features retained = 15, ε = 0.1) was able to predict age comparable to, but worse than, the linear SVR predictor [RBF SVR: ŷ=0.35x+29, *R*^2^ = 0.188, *p*-value < 1 × 10^−3^, (null hypothesis of no correlation or zero slope)]. The node weights were computed in the same way as for the linear SVR case (see Figure 11), and the highest weight nodes are listed in Table 6.

**Table 6:** **Nodes for the RBF SVR predictor**.

ROI index	ROI
23	L_ant_insula
26	M_mFC
44	L_parietal_1
66	R_parietal_3
69	L_parietal_7
74	L_precuneus_1
77	L_IPL_1

However, we use the linear SVR predictor for feature and node significance output since weights extracted from the linear SVR have a direct proportionality between absolute weight and significance in variable prediction. The same cannot be said about the RBF SVR weights, which are not as readily interpreted.

## Discussion

In the present study, we examined the ability of a SVM to classify individuals as either young or old, and to predict age solely on their rs-fMRI data. Our aim was to improve the discriminatory ability and accuracy of the multivariate vector machine method by parameter tuning and feature selection and also output interpretable discriminating features.

Support vector machine classification (using temporal correlations between ROIs as input features) of individuals as either children or adults was found 91% accurate in a study by Dosenbach et al. (2010), and our 84% accurate age classifier is in agreement with these results. This shows that a SVM classifier can be successfully applied to rs-fMRI functional connectivity data with appropriate feature selection and parameter tuning. Our linear SVM classifier’s performance was comparable to that of the RBF SVM, and only slightly more accurate. One advantage of the linear SVM classifier over the RBF classifier, used by Dosenbach et al. (2010) for feature interpretation, is that the weights extracted from the linear classifier have a direct relationship between absolute weight and the classifier contribution. The RBF classifier weights are more difficult to interpret.

Although age classification was very significant (*p*-value < 1 × 10^−7^), gender classification (*p*-value < 0.17) was not. This could be due to the lack of significant differences between resting male and female functional connectivity. A recent study by Weissman-Fogel et al. (2010) found no significant differences between genders in resting functional connectivity of the brain areas within the executive control, salient, and the default mode networks. The performance of our classifier is consistent with this result and suggests that functional connectivity may not be significantly different between genders. This also provides confirmation that the SVM method classification is specific to aging and not other characteristics in this group of individuals such as gender.

We found that the SVM method predicted subject age on a continuous scale with relatively good performance. A perfect predictor has a linear regression fit of ŷ=1x+0, that is, for a given age, *x*, the SVR prediction, *y*, matches that age exactly, implying a ŷ=1x+0 fit with *R*^2^ = 1. The closer the slope of the regression line approached one, and the closer the *R*^2^ value approached one, the better the performance of the predictor was considered to be. The *R*^2^ value is a measure of the proportion of variability of the response variable (predicted age) that is accounted for by the independent variable (true age), so an *R*^2^ of 0.419 (linear SVR) reveals that a substantial portion of the variability in the predicted age is accounted for by the subject age.

From the linear regression plot (Figure 6**)** it appears that the younger subjects are overestimated in their predicted age and the older subjects are underestimated in their predicted age. The subjects around age 40–50 are estimated accurately. For this regression fit (ŷ=0.5x+23),ŷ (the predicted age) ranges from around 30 (when *x* = 20) to around 80 (when *x* = 90) so the predicted age range is smaller than the actual age range – this occurrence may be due to similar connectivity maps of ages in a small range (age 25–30 for example). This difficulty in accurately distinguishing subjects within a small age range could suggest non-significant age-related inter-subject differences in functional connectivity of subjects in small adult age ranges.

**Figure 12:**
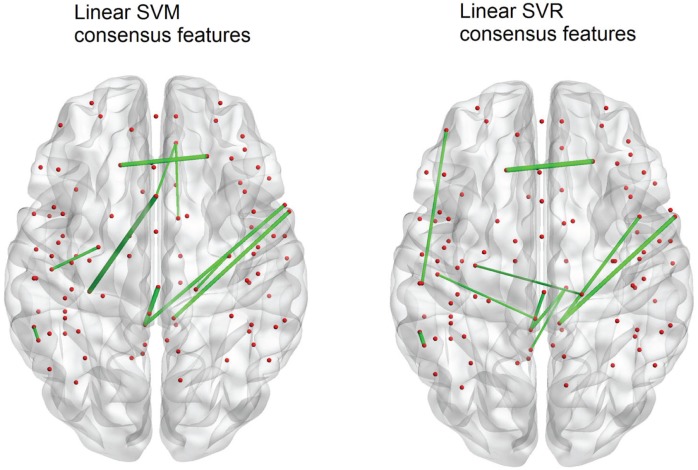
**A comparison of the 10 top consensus features for SVM and SVR**. Each connection thickness is proportional to the feature weight. Overlap of features indicates an agreement for both age classification and prediction techniques.

**Figure 13:**
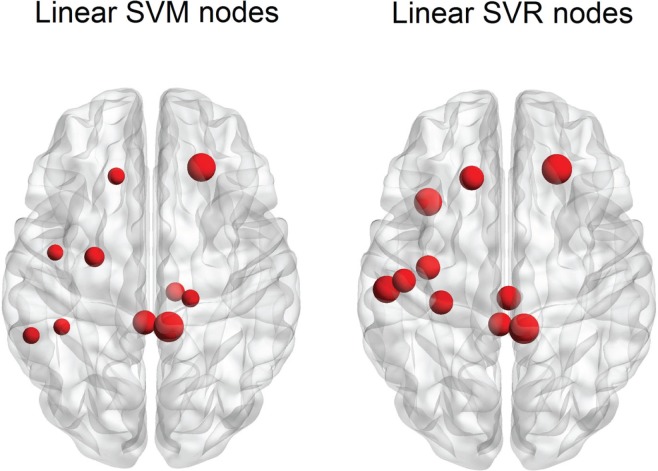
**A comparison of the 10 top nodes for SVM and SVR**. Each node size is proportional to the feature weight. Overlap of nodes indicates an agreement for both age classification and prediction techniques.

The SVM method allows for detection of the most influential features and nodes which drive the classifier or predictor. We utilized this approach to find the “connectivity hubs,” or nodes with the most significant features that influenced age classification. Tables 3 and 5 reveal the 10 most influential nodes for the linear age SVM classifier and for the linear SVR predictor, respectively. Four out of the 10 most influential nodes are present in both methods: R_precuneus_1, R_sup_frontal, L_precuneus_1, and L_sup_frontal (see Figures 12 and 13). There is a similar degree of agreement between the RBF SVR nodes and the linear SVR nodes: L_precuneus_1, L_parietal_1, R_parietal_3, and L_IPL_1 are in both methods. This agreement between classifier and predictor methods suggests that the connectivity of these nodes provides discriminatory information with respect to age differences with some independence of choice of method.

Of note is the difference in distributions of the node weights for the linear SVM and linear SVR methods (Figures 5 and 10). The SVM result seems to have only a few high valued nodes with many quite small valued ones, indicating a more abrupt distribution. The SVR node weight values are distributed more uniformly, with high valued nodes, middle valued, and low valued ones occurring frequently. This could be attributed to the difference in the number of top features retained by the two methods. Since features were projected into their respective nodes and the SVM had 100 features retained while the SVR had 298, the distribution of the SVR node values seemed more uniform.

The improvement in accuracy due to the reduction of the dimension of the feature space, in general, reveals that the classification performance is related to the number of features used and the “quality” of the features used. Our work, using the *t*-test feature filter method for SVM and the correlation feature filter method for SVR as well as the method for parameter selection, shows that SVM classifiers and SVR predictors can achieve high degrees of performance.

The growing number of imaging-based binary classification studies of clinical populations (autism, schizophrenia, depression, and attention-deficit hyperactivity disorder) suggests that this is a promising approach for distinguishing disease states from healthy brains on the basis of measurable differences in spontaneous activity (Shen et al., 2010; Zhang and Raichle, 2010). In addition, several recent studies have demonstrated that the rs-fMRI measurements are reproducible and reliable in young and old populations (Shehzad et al., 2009; Thomason et al., 2011; Song et al., 2012) so a brief resting MRI scan could provide valuable information to aid in screening, diagnosis, and prognosis of patients (Saur et al., 2010). Our own work supports the results that rs-fMRI data contain enough information to make multivariate classifications and predictions of subjects. As the amount of available rs-fMRI data increases, multivariate pattern analysis methods will be able to extract more meaningful information which can be used in complement with human clinical diagnoses to improve overall efficacy.

## Conflict of Interest Statement

The authors declare that the research was conducted in the absence of any commercial or financial relationships that could be construed as a potential conflict of interest.

## Footnotes

^1^www.nitrc.org/projects/fcon_1000

^2^http://afni.nimh.nih.gov/afni

^3^www.fmrib.ox.ac.uk/fsl

^4^http://www.nitric.org/projects/fcon_1000

^5^http://www.nitrc.org/projects/bnv
